# Recent Advancements in Polymer/Liposome Assembly for Drug Delivery: From Surface Modifications to Hybrid Vesicles

**DOI:** 10.3390/polym13071027

**Published:** 2021-03-26

**Authors:** Vincenzo De Leo, Francesco Milano, Angela Agostiano, Lucia Catucci

**Affiliations:** 1 Department of Chemistry, University of Bari, Via Orabona 4, 70126 Bari, Italy; angela.agostiano@uniba.it; 2 Istituto di Scienze delle Produzioni Alimentari (ISPA), Consiglio Nazionale delle Ricerche (CNR), S.P. Lecce-Monteroni, Ecotekne, 73100 Lecce, Italy; francesco.milano@cnr.it

**Keywords:** liposomes, polymers, liposome surface modification, hybrid vesicles, physicochemical stability, encapsulation efficiency, drug release profile, mucopenetrating/mucoadhesive properties, stimuli-responsive properties, versatile targeting platform

## Abstract

Liposomes are consolidated and attractive biomimetic nanocarriers widely used in the field of drug delivery. The structural versatility of liposomes has been exploited for the development of various carriers for the topical or systemic delivery of drugs and bioactive molecules, with the possibility of increasing their bioavailability and stability, and modulating and directing their release, while limiting the side effects at the same time. Nevertheless, first-generation vesicles suffer from some limitations including physical instability, short in vivo circulation lifetime, reduced payload, uncontrolled release properties, and low targeting abilities. Therefore, liposome preparation technology soon took advantage of the possibility of improving vesicle performance using both natural and synthetic polymers. Polymers can easily be synthesized in a controlled manner over a wide range of molecular weights and in a low dispersity range. Their properties are widely tunable and therefore allow the low chemical versatility typical of lipids to be overcome. Moreover, depending on their structure, polymers can be used to create a simple covering on the liposome surface or to intercalate in the phospholipid bilayer to give rise to real hybrid structures. This review illustrates the main strategies implemented in the field of polymer/liposome assembly for drug delivery, with a look at the most recent publications without neglecting basic concepts for a simple and complete understanding by the reader.

## 1. Introduction

**Scheme 1:**
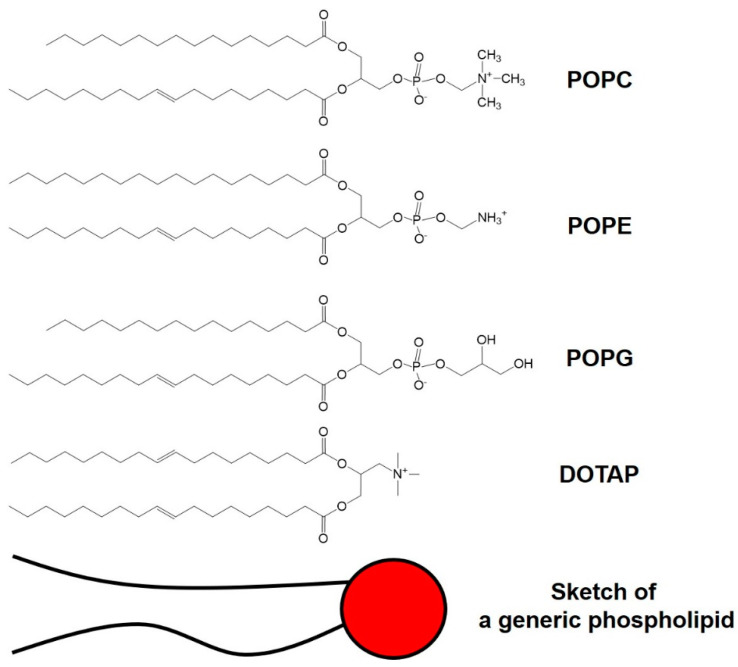
Chemical structures of most commonly used (phospho)lipids for liposome preparation. From top to bottom: The zwitterionic 1-palmitoyl-2-oleoyl-phosphatidylcholine (POPC), principal constituent of natural membranes; the zwitterionic and fusogenic 1-palmitoyl-2-oleoyl-phosphatidylethanolamine (POPE); the anionic 1-palmitoyl-2-oleoyl-phosphatidylglycerol (POPG); the cationic lipid 1,2-dioleoyl-3-trimethylammonium-propane (DOTAP). Bottom: Schematic drawing of a generic phospholipid with the two alkyl chains depicted as black wires and the polar head represented by a red circle.

Since their appearance at the limelight of science, liposomes have offered researchers a great variety of application possibilities despite the relative simplicity of their preparation. The lipid molecules (mostly phospholipids, Scheme 1) of which they are made behave like building blocks that auto-assemble in a supramolecular architecture ordered on spherical shapes, once they are dispersed in an aqueous environment. These lipid molecules, with the hydrophobic region paired to exclude water, form double layers that close in on themselves, enclosing part of the solvent in which they are dispersed, similarly to biological membranes [1,2,3]. Their peculiar architecture and structural analogy with biological membranes have given impetus to studies on membrane fusion, on the reconstitution of integral membrane proteins, on membrane compartmentalization, and finally, on protocells and the origins of life [4]. Moreover, the structural versatility of liposomes allow the design of vesicles of any size, degree of lamellarity, and composition for the most varied applications. The possibility of loading liposomes with molecules of different polarities both in their aqueous lumen and in the phospholipid bilayer, and the possibility of decorating them with a variety of molecules to gain peculiar properties, soon opened the way for their use in the field of drug delivery and targeting. An extraordinary scientific production has been dedicated to the development of liposomal carriers for the topical or systemic delivery of drugs and bioactive molecules, with the possibility of increasing their bioavailability and stability, and modulating and directing their release, while limiting the side effects at the same time. Today, liposomes for tumor targeting, gene therapy, genetic vaccines, immunomodulation, photodynamic therapy, transdermal applications, and many more represent a concrete reality in real-time clinical applications [5,6]. Furthermore, the versatility of liposomes is such that they can bioconjugate and increase the biocompatibility even of hard materials, both nanostructured (such as quantum dots, carbon nanotubes, and gold nanoparticles) and inorganic-based surfaces (such as titanium and silica), for the production of fluorescent and magnetic probes [7,8,9,10] or medicated surfaces for prostheses or other applications [11].

However, liposomes are not free from defects. Their stability over time is limited, and in the absence of precautions, they show a tendency to coalesce and sediment. In the presence of biological fluids, plasma proteins interact with the surface, limiting their in vivo circulation lifetime [12]. The payload that can be retained is limited and the hydrophilic one can diffuse unwanted across the membrane. Finally, liposomes are characterized by a lack of site-specificity, as well as low targeting abilities [5]. Therefore, the liposome preparation technology soon took advantage of the possibility of improving vesicle performance using both natural and synthetic polymers.

Polymers can easily be synthesized in a controlled manner over a wide range of molecular weights and in a low dispersity range. They are more stable than lipid molecules and less prone to undergoing oxidative degradation phenomena. Like the lipids used for the preparation of liposomes, amphiphilic polymers can be prepared with the most varied block structures, and therefore, they are able to intercalate in the lipid bilayer. Their properties are widely tunable and therefore allow the low chemical versatility typical of lipids to be overcome [13].

**Figure 1:**
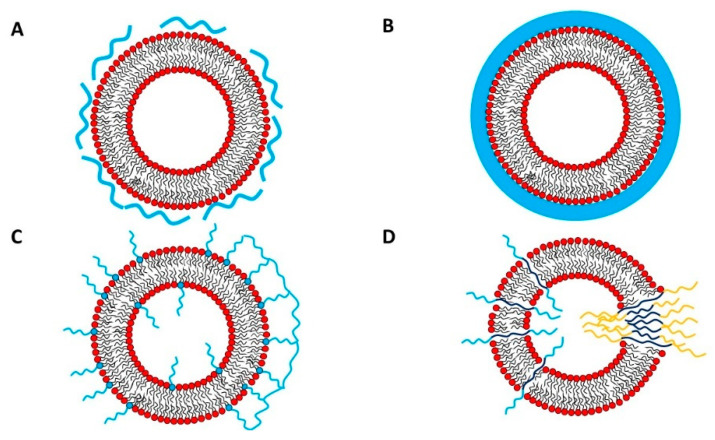
Polymer/liposome assembly. (**A**) Hydrophilic polymers are physically adsorbed onto liposome surface; (**B**) polymers are polymerized or undergo a phase change, trapping the vesicles inside; (**C**, **left**) polymers are covalently bonded to the polar head of phospholipids; (**C**, **right**) polymerizable monomers are reticulated to form a network surrounding the vesicles; (**D**, **left**) amphiphilic polymers are homogeneously distributed in the bilayer or (**D**, **right**) segregated in separate domains.

There are a variety of ways in which polymers of various kinds can become part of the architecture of liposomes (Figure 1). Hydrophilic polymers can coat the surface of the vesicles by physical adsorption (Figure 1A). Neutral polymers (poly-vinyl pyrrolidone (PVP), poly-vinyl alcohol (PVA), etc.) can adsorb by hydrogen bonds to the vesicle surface rich in hydroxyl and oxo groups [14], and the ionic ones (chitosan, polylysine, etc.) can interact by electrostatic interaction with the bilayer charged by ionic lipids or other additives [15,16]. In addition, by suitably choosing oppositely charged polyelectrolytes, it is possible to realize a multilayer shell by the layer-by-layer electrostatic deposition method [17].

In other circumstances, a polymer can be polymerized starting from its monomers in the presence of liposomes, thus trapping vesicles within their structure (Figure 1B) [18]. The same result can be obtained with polymers whose solubility depends on the pH, by adding them to a liposome suspension in soluble form and then inducing their solidification by appropriately varying the pH [19,20]. Alternatively, liposomes can be included in a hybrid hydrogel matrix of polysaccharides [21].

A very popular way to coat liposomes with polymers is to covalently bond polymers to the polar head of phospholipids. Some of these modified phospholipids are commercially available or can be synthesized according to specific needs. This is the case of polyethyleneglycol(PEG)ylated and polyglycerol(PG)ylated lipids, respectively. They can be easily dispersed in the lipid blend during vesicle preparation. After hydration of the lipid film, these modified lipids self-assemble in the bilayer, with the lipid part acting as a hydrophobic anchor in the lipid palisade and the hydrophilic polymeric portion facing the aqueous part [22,23] (Figure 1C, left). Alternatively, polymerizable monomers can be linked to lipid molecules or to cholesterol, to induce their polymerization to form a network surrounding the vesicles (Figure 1C, right) [24]. It is also possible to crosslink the hydrophobic tails of the lipids within the liposomal bilayers [25].

A mixture of lipids and amphiphilic polymers can be used to give rise to hybrid vesicles, in order to obtain structures with intermediate physical properties between the two amphiphiles (Figure 1D). Many block copolymers are commercially available, and others can be synthesized to fit specific experimental needs. The preparation of hybrid vesicles does not involve particular changes in the traditional protocols for the preparation of liposomes, and the copolymers are generally added to the lipid blend in the first stage of vesicle preparation. The type of structure that can be obtained depends on the compatibility between the lipid and polymeric building blocks in terms of dimensions and chemical–physical properties, as well as the polymers/lipids ratio. It is therefore possible to obtain very different types of vesicles, in which the limit forms have the polymeric molecules homogeneously distributed in the bilayer (Figure 1D, left) [22,26,27] or segregated in separate domains (Figure 1D, right). Although this last configuration has mainly been observed and studied in micrometric giant vesicles that have limited applications in the field of drug delivery [26,27], examples relating to nanostructured vesicles have also been reported [28].

Although the reasons for using polymers in liposome preparations are very numerous, they can be grouped into the following macro categories, all aiming to overcome some classic limitations of first-generation liposomes or endow them with specific properties and potentials:

- Improving the physicochemical stability, the stealth properties, and the residence time in biological fluids
- Improving the encapsulation efficiency, reducing payload leakiness, and modulating drug release profile
- Conferring mucopenetrating/mucoadhesive properties
- Conferring stimuli-responsive properties
- Providing a versatile targeting platform

Obviously, that proposed is only one of the possible classifications, and does not come without a certain degree of arbitrariness. In fact, each property induced by polymers on liposomes inevitably borders on or partially overlaps with other properties. Decorating a vesicle with a hydrophilic polymer such as PEG means stabilizing it sterically at the same time, giving it stealth and mucopenetrating properties, with the possibility of also introducing useful functions for targeting purposes. In any case, in this review, the various strategies implemented in the field of polymer/liposome assembly for drug delivery were summarized and illustrated, with a look at the most recent publications without neglecting basic concepts for a simple and complete understanding by the reader.

## 2. Polymer/Liposome Assembly to Improve Physicochemical Stability, Stealth Properties, and Residence Time of Vesicles in Biological Fluids

**Figure 2:**
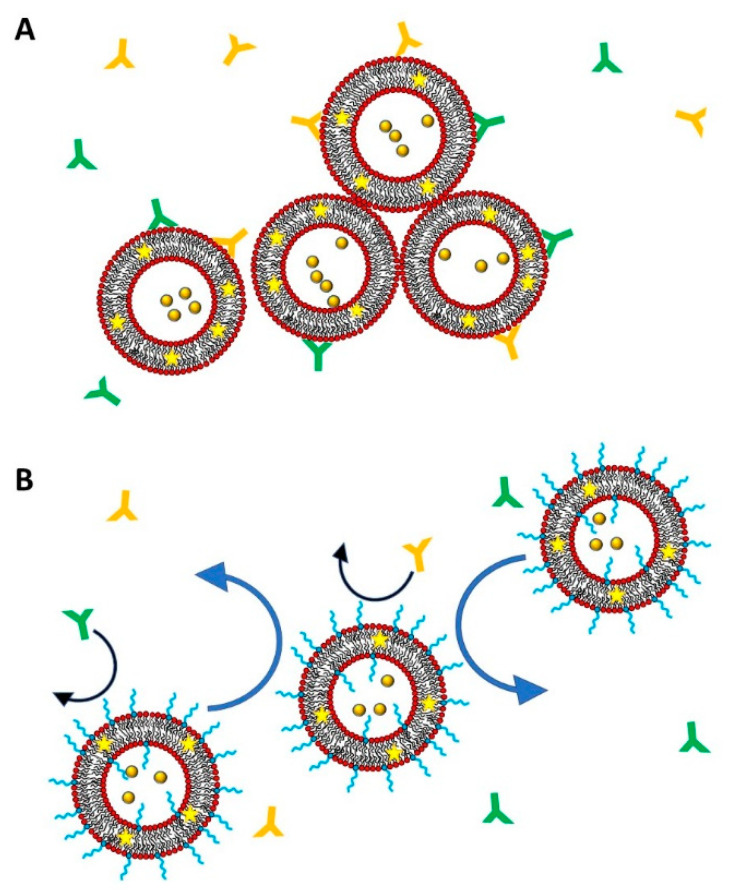
Scheme representing the mechanism for increased circulation time of PEG-decorated liposomes. (**A**) Conventional liposomes, bearing hydrophilic (orange balls) and hydrophobic (yellow stars) loads, aggregated and attacked by plasma proteins (Y-shaped antibodies in yellow and green); (**B**) stealth liposomes with PEG functionalization (cyan “hairs”) whose surface cannot be reached by plasma proteins.

Liposomes present important advantages such as biocompatibility, biodegradability, and the ability to encapsulate hydrophilic, hydrophobic, and amphiphilic compounds. However, one of the major drawbacks of conventional liposomes is their rapid clearance from the bloodstream [29,30]. To overcome this drawback, surface modification of the liposomes has proven to be a successful strategy to improve the stability and half-life of liposomes in the bloodstream. In particular, hydrophilic polymers, such as polyethylene glycol (PEG), are used as surface coatings in order to extend the liposome blood circulation half-life from a few minutes to several hours [31]. The mechanism of improving their circulation time was attributed to steric hindrance induced by PEG that prevents their aggregation and inhibits the absorption of plasma proteins and reticuloendothelial system uptake [32,33] (Figure 2).

The steric stabilization conferred by PEG is influenced by the size of the liposomes, as well as to the extension of PEGylation. It was reported that for sizes higher than 275 nm, the stealth property of PEG-liposomes is significantly compromised [34], and that extensive PEGylation can also cause the inhibition of cellular uptake [33].

Moreover, the chemical and structural features of PEG derivatives influence the stability and stealth properties of PEG-decorated liposomes. Mastrotto et al. evidenced that in vivo, long linear, or branched PEGs chains are less efficient in improving the stealth features of liposomes compared to short PEG chains. This behavior was attributed to the lower possible resistance of long/branched PEGs to shear stress arising during blood circulation [35]. Recent studies also exploited the possibility of the PEG on PEGylated liposomes of binding ligands for active targeting, thus combining longevity and targetability for drug delivery into specific tissues [36].

Taking into account the properties of the PEG listed above and the fact that it is soluble in both polar and nonpolar solvents, and can be eliminated from the body through a combination of renal and hepatic pathways [37], it is possible to understand why PEG is an effective polymer coating for pharmaceutical application.

PEG-liposomes, however, have been found to induce immunogenic responses, which can lead to hypersensitivity reactions [38,39,40], and to the accelerated blood clearance phenomenon, which accounts for the rapid systemic clearance of PEGylated nanocarriers upon repeated administrations [41]. Therefore, although PEG remains the gold standard for the steric protection of liposomes, many studies aim to identify other polymers that can confer stability and long circulating properties to liposomes.

Earlier studies described long-circulating liposome preparation using poly[*N*-(2-hydroxypropyl) methacrylamide] [42], polyvinyl alcohol [43], and L-amino-acid-based biodegradable polymer–lipid conjugates [44]. Studies on the influence of liposome charge and polymer molecular mass evidenced that opsonins with different molecular masses might be involved in the clearance of liposomes [45].

Recently, liposomes modified with polyglycerol have also been proposed as an alternative to PEGylation. The use of polyglycerol, in fact, prevents induction of the accelerated blood clearance phenomenon upon repeated administration. Moreover, hyperbranched polyglycerol exhibiting multiple hydroxyl groups at their ends appeared to mediate a stronger cellular internalization into macrophages [41].

Superhydrophilic zwitterionic polymers such as poly (carboxybetaine) (PCB) can stabilize liposomes as an alternative to PEG. In addition, PCB-modified liposomes exhibited good retention of the hydrophilic drug and long blood circulating characteristics in vivo without the need to add cholesterol to the lipid formulation [46]. On the other hand, a novel lipid/poly-phosphocholine conjugate can stabilize the liposomes against aggregation similarly to PEG and allow them to act as very efficient lubricating elements, readily attaining superlubric performance, useful in potential biomedical applications [47].

Pluronic F127 (PF127) is another polymer used as an alternative to the most traditional PEG for liposome coating. PF127 is a triblock nonionic surfactant largely used as a food additive and is approved as a pharmaceutical component for cancer drugs. It has a high circulation time, high bioavailability, and can stabilize liposome preparations. Stable dipalmitoyl phosphatidylcholine liposomes coated with an PF127 copolymer were obtained for hypericin loading and delivery [48]. In this study, the liposome preparation was kept stable by the copolymer for 6 months in the solid state and up to 20 days for the nondry formulation. In addition, thermal stability of the formulation was observed up to 50 °C. In addition, the modification of liposomes with PF127 can enhance their mucus penetration and cellular uptake [22,49] (see Section 4). PF127-covered liposomes also show the more efficient delivery of coumarin 6 to enterocytes than unmodified liposomes [50].

**Figure 3:**
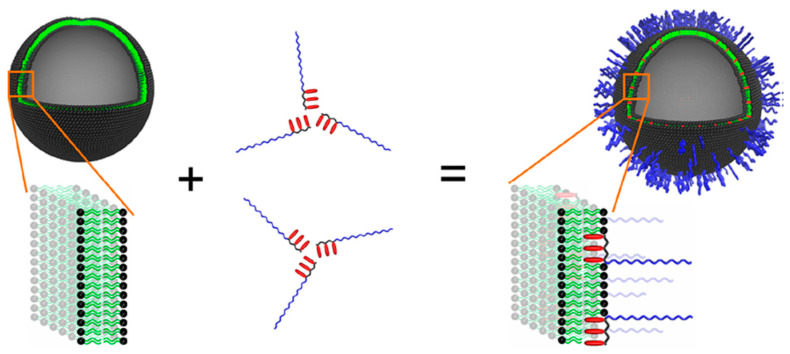
Hybrid stealth liposomes stabilized by cholesteryl-functionalized block copolymers. The cholesteryl moieties (red sticks) insert into the bilayer while the tails (in blue) protrude toward the aqueous phase, protecting the liposomes from coalescence and plasma proteins. Reproduced with permission from [52]. Copyright © 2018 American Chemical Society.

Other useful copolymers are 2-methacryloyloxyethyl phosphorylcholine and n-butyl methacrylate (PMPC-co-BMA) or the latest 2-(methacryloyloxy)ethyl phosphorylcholine and methacrylated polyhedral oligomeric silsesquioxane that can interact with the liposome surface and enhance its stability in physiological conditions [51]. Recently, novel cholesteryl-functionalized block copolymers as molecular stabilizers for stealth liposome preparation have been proposed by Kenneth et al. [52]. The authors observed that the employed block copolymers offer resistance to micellization through the insertion into the lipid bilayer of multiple cholesteryl moieties per molecule, with a minimum number of such moieties per molecule required for effective copolymer insertion into the bilayer and liposome stabilization (Figure 3).

Hyaluronic acid (HA)-coated liposomal formulations have also been studied. HA is a natural negatively charged linear hydrophilic polysaccharide, composed of the repeating glucuronic acid and *N*-acetyl-d-glucosamine disaccharide units linked by alternating β-1,4 and β-1,3 glycosidic bonds. The biocompatibility, biodegradability, nontoxic, and nonimmunogenic nature make HA a suitable alternative to PEG [53]. Different studies indicated that the hydrophilic coating provided by HA prevents opsonin adsorption to the liposome surface and increases drug circulation, as well as the affinity binding to tumor recognition sites [41,53,54].

Among polysaccharides, chitosan is largely used as a coating material because it is positively charged and readily interacts with the negatively charged liposomal surfaces, ensuring a firm coating that enhances liposome stability, with respect to both size and drug loading, in the biological fluid [33,55,56]. To increase the resistance of chitosan-based systems to the harsh gastrointestinal environment, modifications of their surface to obtain multilayered or multivesicular carriers have been reported [54,55,56,57,58]. Multivesicular carriers created by coating liposomes with chitosan followed by crosslinking with biocompatible β-glycerophosphate were reported [59]. The possibility of functionalizing chitosan has made it possible to obtain copolymers such as PEG-chitosan that stabilizes liposomes, encapsulating the novel prodrug of doxorubicin modified by stearoyl-spermine [60].

Another polysaccharide used for the stabilization of liposomal systems is starch given its great availability, biocompatibility, and biodegradability [61]. For example, Nahar et al. prepared stable starch-coated magnetic liposomes that resulted in a promising inhalable carrier for accumulation of the fasudil drug in the pulmonary vasculature [62], while Salem et al. developed an oral starch-liposome formulation that increased the stability and tolerability of sodium alendronate [63].

Increased alendronate sodium oral bioavailability was also achieved by covering the liposomes with Eudragit L100 [64]. Eudragits are pH-sensitive methacrylic acid copolymers, which are of great importance especially in the oral delivery of bioactive molecules. In fact, the main obstacles to efficient oral drug delivery using liposome carriers are the high acidity of the gastric environment, the presence of enzymes, and the mucosal barrier [58] (see Section 4). In particular, Eudragit S100 with increased solubility at pH ≥ 7 is suitable for the development of pH-responsive carriers for colonic drug delivery (see Section 5).

Another study reported a liposome system decorated with proteins and DNA that combine the useful properties of both liposomes and biomacromolecules. In particular, liposomes decorated by crosslinked and glycosylated lactoferrin were stabilized by the protein layer and preserved the antioxidant activity of the encapsulated 7,8-dihydroxyflavone, thus resulting in promising delivery systems for protecting and transporting bioactive components [65]. Lectin-conjugated liposomes were also prepared as biocompatible, bio-adhesive drug carriers [66]. On the other hand, functionalizing liposomes with DNA has produced a diverse range of hybrid materials useful in drug delivery. For example, liposomes densely functionalized with DNA hindered the degradation of the conjugate by serum proteins, increasing the stability in biological environments and cellular uptake [67]. Baumann et al. prepared lipid vesicles coated and stabilized by a semirigid DNA network, based on the connection of three-arm branched DNA junctions inspired by the structure of clathrin [68]. As the self-assembly of clathrin on biological membranes facilitates the endocytosis process, materials inspired by its ordered structural appearance are of great interest. Combining this aspect with the possibility to functionalize the DNA, liposomes prepared by Baumann et al. are particularly promising for drug delivery application.

## 3. Polymer/Liposome Assembly to Improve Encapsulation Efficiency, Reducing Payload Leakiness and Modulating Drug Release Profile

**Figure 4:**
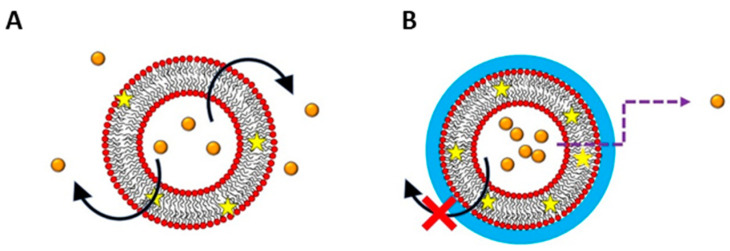
(**A**) Conventional liposome-bearing hydrophilic (orange balls) and hydrophobic (yellow stars) loads. The hydrophilic cargo tends to cross the bilayer leaking to the external aqueous phase. (**B**) Polymer-coated liposome with reduced bilayer permeability and higher cargo retention, enabling sustained and controlled drug delivery.

In addition to the steric stabilization, the grafting of polymers into the lipid bilayer increases the hydrophilicity of the liposome and their subsequent stability in an aqueous environment [69], and influences liposome permeability and physicochemical properties such as drug loading and leakage [70] (Figure 4). For example, PEGylated liposomes have been found to possess high drug loading capacities up to 90% [71,72]. Furthermore, the presence of PEG in the liposome composition has been shown to reduce leakage of the entrapped compounds from liposome particles kept in a phosphate buffer solution (PBS) and in fetal bovine serum (FBS) [73]. The membrane permeability of PEGylated liposomes is, in fact, affected by the molecular weight of the PEG molecule, the amount of PEG-lipid conjugates, and the type of linkage between the hydrocarbon chain and PEG chain of PEG-lipid conjugates [74]. In particular, the addition of an amide linkage in a conjugated chain has been shown to reduce the leakage of the entrapped model drug goniodiol without affecting the entrapment efficiency, by decreasing the liposome permeability [73]. In addition, the coverage of liposomal surfaces with layers of natural polymers such as enteric polymers, proteins, and polysaccharides, generally used to protect liposome in the gastrointestinal tract, reduces payload leakiness, enhancing the drug bioavailability [57,64]. For example, the cross-linked chitosan/liposome hybrid system showed a high entrapment efficiency of quercetin [75], while Eudragit S100-coated liposomes improved the site-specific release, preventing drug leakiness [20,57], and poly-electrolyte-stabilized liposomes loaded with doxorubicin showed 4–6-fold-enhanced oral drug bioavailability with respect to conventional liposomes [55].

The drug release rate depends on the composition of liposomal membranes, such as the type of fatty acid acyl chains of phospholipids and percentage of cholesterol, that affects the rigidity of the carrier membrane [76]. However, by combining the relatively compact lipid bilayer interior with a mobile steric surface barrier on the vesicle surface, one should obtain lipid vesicles that are optimally suited for both the long-term circulation in the blood and for the sustained drug release under physiological conditions [77]. Therefore, liposomes for the sustained drug release were made by modulating the properties of the bilayer (by adding, for example, particular components such as glycolipids, phosphatidylinositol, and monosialoganglioside [78]), or by the surface modification of the liposomes with sialo-glycopeptides or PEG [77]. However, very recently, it has been shown that in individuals with anti-PEG antibodies, a rapid release of drugs by PEGylated liposomes occurs. In fact, it has been shown that anti-PEG IgG and IgM antibodies bind to PEG molecules on the surface of the liposomal doxorubicin PEG coating (Doxil, Doxisome, LC-101 and Lipo-Dox), with the consequent activation of the complement, formation of a complex of membrane attachment in the liposomal membrane, and the rapid release of the encapsulated drug of up to 40% from the liposome [79].

In addition, hydrogels based on both natural and synthetic polymers can be used as depots for bioactive agent-loaded liposomes, for slow drug release. Among others, polymer hydrogels based on fibrin, chitosan, alginate, dextran, Carbopol, and polyvinyl alcohol have been used for this purpose. In this way, a sustained release of drugs loaded with liposome in a polymeric-based depot system offers the possibility of reducing the dosing frequency and the side effects [80]. In this context, the modulated release of a model hydrophilic drug from liposomes entrapped in chitosan/gelatin hydrogels obtained by double crosslinking with glutaraldehyde and sodium sulphate/sodium tripolyphosphate has been described [81]. A release-controlling liposome-modified hydrogel as an artificial scaffold for promoting the angiogenesis and osteogenesis in bone regeneration was recently designed. A photo-crosslinkable gelatin derivative (GelMA) was combined with a drug-loaded liposome, and the ability to control the phased release was observed in the composite hydrogel, including the early release of the hydrophilic drug (deferoxamine), mid-term release of the bioactive macromolecule (bovine serum albumin and bone morphogenetic protein 2), and long-term release of the liposoluble medicine (paclitaxel) [82].

## 4. Polymer/Liposome Assembly to Confer Mucopenetrating/Mucoadhesive Properties to Vesicles

**Figure 5:**
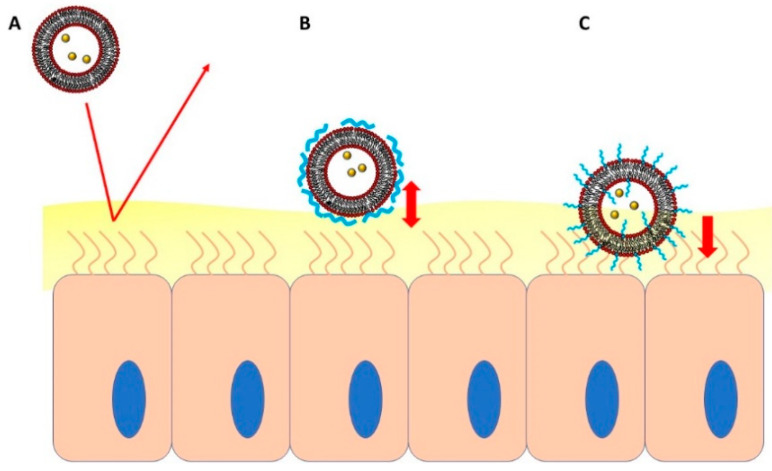
Scheme of a generic tissue formed by several cells (in orange with blue nuclei) covered by mucus (in yellow). (**A**) Conventional liposome unable to cross the mucosal barrier; (**B**) liposome covered by polymer, increasing the mucoadhesive properties of the vesicle; (**C**) liposome decorated with polymer conferring mucopenetrating properties to the vesicle.

Mucus is a complex aqueous gel layer that covers the mucosal membranes of the respiratory tract, the gastrointestinal tract, the reproductive tract, and also the ocular surface. Its composition depends on the anatomical site where it is produced and on the health conditions of the underlying epithelium, but it mainly comprises water and mucin fibers, lipids, salts, proteins, as well as sloughed cells, bacteria, and various cellular debris [83]. The viscoelastic properties of the mucus very often constitute a formidable obstacle for drug delivery systems that have to cross it before delivering the drugs to their target (Figure 5A). Nanostructured carriers have been shown to have the ability to cross the dense network of mucin more effectively than large ones. Therefore, first-generation phospholipid/cholesterol liposomes of suitable size (<100 nm) are ideal candidates for this purpose. However, it is possible to use a number of natural and synthetic polymers to enhance the transport capacities of liposomes and also to increase their stability in these harsh conditions [84]. Two opposite strategies are possible to increase the drug delivery performance of liposomes when mucus is an obstacle, decorating the surface of the vesicles with suitable polymers. The first way is to increase the mucoadhesive properties of the vesicles, so that the liposomes can adhere to the mucus layer to increase the residence time of the incorporated drugs and therefore to improve the contact with the absorption membranes (Figure 5B). On the contrary, the second way involves making the liposomes mucopenetrating, that is, the ability to diffuse more deeply through the mucus layer and therefore more effectively reach the underlying epithelium (Figure 5C) [84].

In vitro tests showed that positively charged but uncoated liposomes already exhibit mucoadhesion; therefore, this property is believed to be driven primarily by electrostatic interactions with the negatively charged glycoproteins that make up mucus [85,86]. However, positively charged naked liposomes show limited biocompatibility, and therefore, coverage with polymers is usually preferred. Surface-modified liposomes can be made by coating with both natural or modified mucoadhesive polysaccharides such as alginate, chitosan, pectin, or with synthetic polymers such as Eudragit and Carbopol [21,87,88,89]. Natural polysaccharides are extracted from plants, algae, and the exoskeleton of some crustaceans. These natural polymers are biocompatible and generally safe to use, and exhibit strong mucoadhesive properties through electrostatic interactions or hydrogen bond formation [84,90]. It is also possible to increase the mucoadhesive properties of these polymers with appropriate derivatizations at the –OH and –COOH groups. Chitosan, a cationic polysaccharide, is perhaps the most popular of the polymers used in association with liposomes to obtain mucoadhesive vesicles. Adamczake and co-authors prepared polysaccharide-coated liposomes with a positive, negative, or neutral charge for drug delivery to the oral cavity. Several polysaccharides were used for coating vesicles: Alginate and low-ester pectin (both hydrophilic and negatively charged), chitosan (hydrophilic and positively charged), and hydrophobically modified ethyl hydroxyethyl cellulose (amphiphilic and neutrally charged). The mucoadhesion properties were studied using an in vitro method, allowing the vesicles to interact with a mucus-producing confluent HT29-MTX cell-line. Positive chitosan-coated liposomes showed the best mucoadhesive properties, although the chitosan-coated systems showed lower biocompatibility than the uncoated systems. Alginate-coated liposomes proved to be an attractive alternative in the treatment of chronic diseases of the oral mucosa, thanks to their higher mucosal biocompatibility, specific mucin interactions, and moderate mucoadhesion properties [91]. Natural antioxidants with antimicrobial activity such as resveratrol and curcumin have also been incorporated into chitosan-coated liposomes, for example, for vaginal delivery. The increased bioadhesiveness and the good mucus permeation capabilities make chitosan-coated liposomes suitable systems for the topical treatment of vaginal inflammation and infections [92,93].

Many chitosan derivatives have been recently prepared to enhance its mucoadhesiveness and overcome its limited solubility in water at neutral and basic pH [94]. Zhao and collaborators used chitosan glycol to coat liposomes loaded with Sorafenib, thus overcoming the limitations of native chitosan, which has a very low aqueous solubility above pH 6.5. A second layer of Eudragit S100 was then added to protect the glycol chitosan-coated liposomes from the gastric environment and release them at pH ≥ 7. This layer-by-layer coverage strategy allowed the increase in the cellular uptake of Sorafenib in Caco-2 cells with moderate toxicity [95]. Al Harthi et al. developed liposomal donepezil HCl dispersed into thiolated chitosan hydrogel for the treatment of Alzheimer’s disease. A disulfide bridge formed by interaction between the thiol groups of modified chitosan and the cysteine groups of glycoproteins in the mucus, improving the mucoadhesive properties of the liposomes. The in vivo results showed that liposome incorporated into chitosan hydrogel significantly increased the blood concentration and the brain content of donepezil compared to the oral tablets, and that thiolated chitosan had the highest mucoadhesive capability [96]. At the same time, thiolated chitosan enables controllable drug release, permeation and enhancement of cell absorption, inhibition of efflux pumps and enzymes, and other useful properties [94].

**Figure 6:**
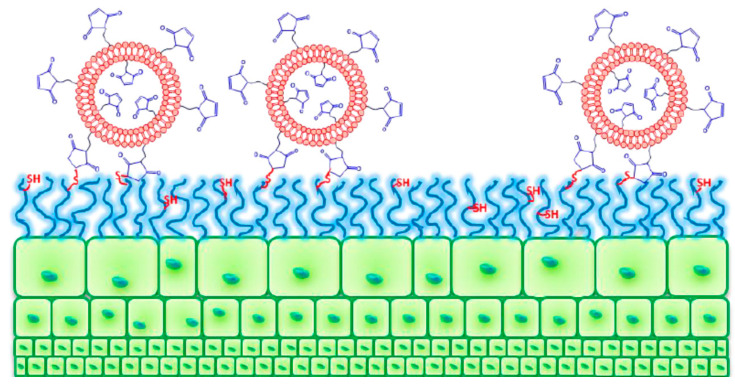
Maleimide-functionalized PEGylated liposomes showing mucoadhesive properties thanks to the covalent linkages with thiol-groups present in mucins. Reproduced with permission from [97]. Copyright © 2018 Elsevier.

Fully synthetic polymers are also useful in increasing the mucoadhesive properties of liposomes. For example, maleimide-functionalized PEGylated liposomes were explored as mucoadhesive vehicles for drug delivery to the urinary bladder. These vesicles exhibited greater retention on mucosal surfaces compared to other tested liposomes and longer drug release properties. Excellent mucoadhesive performance of maleimide-functionalized PEG is due to the ability of the polymer to form covalent linkages with thiol-groups present in mucins [97] (Figure 6).

On the other hand, the polymers used to confer mucus-penetrating properties to the liposomes are nonionic, long-chained, and hydrophilic in nature; that is, they are polymers capable of avoiding most of the weak interactions that allow the adhesion with mucus, namely those of a hydrophobic and electrostatic nature. Polymers such as PEG and Pluronic copolymers are widely employed in liposome modification to improve vesicle diffusion in highly viscoelastic mucus. For example, Jøraholmen et al. developed mucus-penetrating PEG-liposomes containing interferon α-2b for the localized therapy of human papilloma virus infections. Ex vivo penetration studies performed on the vaginal tissue obtained from pregnant sheep showed elevated interferon penetration from PEG-liposomes with respect to the control. In addition, the absence of interaction between the PEG-modified liposomes and mucin was shown [98].

The way in which the polymer decorates the liposome surface can affect its effectiveness. Li et al. investigated the intestinal mucus-penetrating properties of two types of liposomes modified by PF127, i.e., PF127-inlaid liposomes and PF127-adsorbed liposomes. Cellular uptake studies were conducted in Caco-2 cells and analyzed using both confocal laser scanning microcopy and flow cytometry. The diffusion efficiency of the two types of PF127-modified liposomes through intestinal rat mucus was found to be higher than that of unmodified liposomes, but PF127-inlaid liposomes showed a significantly higher cellular uptake with respect to PF127-adsorbed liposomes. In addition, the two types of PF127-modified liposomes seem to have different cellular uptake mechanisms [49].

The polymer molecular weight and surface density on the nanocarrier also affect the mucus penetrating ability, although their real effect should be evaluated case by case [99]. In a very recent paper, Yamazoe et al. investigated the feasibility of densely PEG-modified liposomes for the oral delivery of peptides in an in vitro artificial mucus model. In addition, they compared the oral absorption of these mucus-penetrating vesicles and mucoadhesive liposomes modified with glycol chitosan. The intracellular uptake of both liposomes was evaluated in Caco-2 and mucus-secreting Caco-2/HT29 cultures. The intracellular uptake of PEG-liposomes was unaffected by mucus in the co-culture system, whereas the cellular uptake of glycol chitosan-liposomes was lower. Oral absorption in vivo was higher for densely PEGylated with respect to unmodified liposomes and was PEG-concentration-dependent. In any case, an excessive PEGylation decreased drug blood concentration [100].

**Figure 7:**
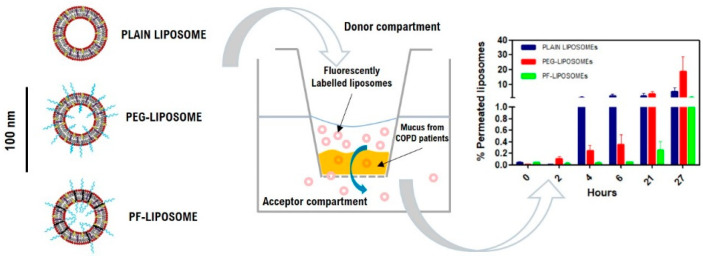
Experimental design to assess the mucopenetrative properties of plain-, PF127-, and PEG-liposomes, using the mucus of chronic obstructive pulmonary disease (COPD) patients. Reproduced with permission from [22]. Copyright © 2018 Elsevier.

Recently, PF127- and PEG-liposomes were prepared for the treatment of chronic respiratory diseases by inhalation, and their ability to penetrate a pathological mucus obtained from chronic obstructive pulmonary disease (COPD)-affected patients was compared. Beclomethasone dipropionate was used as a model drug, and small unilamellar liposomes of about 50 nm and of a surface electric charge close to zero were made by the detergent depletion method. The penetration studies of mucus from COPD patients showed that the PEG-liposomes were the most mucus-penetrating vesicles after 27 h (Figure 7). Both preparations with the two polymers did not cause any effect on bronchoalveolar lavage fluid proteins after aerosol administration in the mouse. However, PEG-liposomes proved to be most valid in terms of penetration through the pathologic sputum, uptake by airway epithelial cells, and safety profile [22].

Finally, liposomes modified with a combination of polymers have been proposed that can perform both the functions of mucus-adhesion and mucus-penetration. Liu et al. developed a mucus adhesion- and penetration-functionalized chitosan-thioglycolic acid-Pluronic F127 (CS-TGA-PF) liposome system for oral delivery of paclitaxel. The prepared liposomes were more stable than the unmodified ones and demonstrated a sustained release of paclitaxel in simulated gastric fluid and intestinal fluid. In addition, CS-TGA-PF liposomes absorbed a three-fold amount of mucin compared with that of unmodified vesicles, which would prolong their residence time on the mucosal surface of the intestinal tract. The intestinal mucus adhesion and penetration efficacy of modified liposomes was studied by observing the intestinal absorption and distribution. The results showed increased liposome uptake by the gastrointestinal mucosa and improved drug intestinal absorption [101].

## 5. Polymer/Liposome Assembly to Confer Stimuli-Responsive Properties to Vesicles

**Figure 8:**
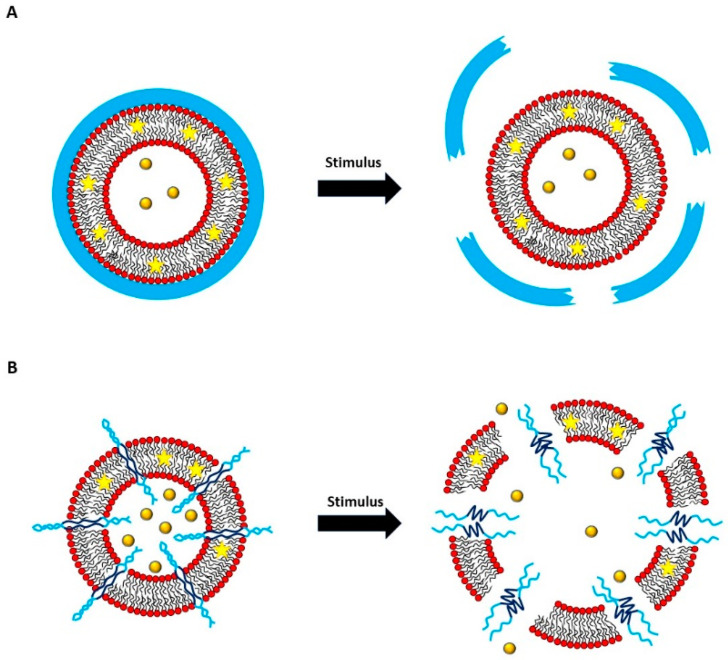
Stimuli-responsive polymeric coatings of liposomes. In (**A**), the stimulus dissolves the protecting coating, while in (**B**), the stimulus destabilizes the bilayer and/or induces the formation of transmembrane channels.

To be strongly effective, liposomes should be able to enter into the target cells as intact structures and release the encapsulated drug in the desired region. To this end, a common practice is to cover or decorate the liposomes with particular functional groups and coating materials to extend the systemic circulation time and enhance the penetration capability toward the target cells. Near the target cell or after entrance, usually through the endocytosis (more often pinocytosis) mechanism, the coating should be amenable, under the action of particular external stimuli, to be dissolved or detach from the liposome surface (Figure 8A), leading ultimately to the drug release to the external medium. Alternatively, when liposomes are internalized in the endosomes, the local acidic pH can induce a conformational change of the coating polymer that can result in (i) the formation of transmembrane channels (Figure 8B) or (ii) the collapse of the coating polymer that destroys the liposome bilayer. Both mechanisms lead to the release of the encapsulated material, enhancing its intracellular bioavailability, especially when the drug is metabolized in lysosomes.

Temperature and pH are widely studied triggering stimuli as they often differ from physiological values in pathological areas [102,103]. For example, lower pH values are typical for inflammation areas, solid tumors, and heart or brain tissues injured by ischemia [104]. pH-Sensitive liposomes (PSL) made of 1,2-diacyl-sn-glycero-3-phosphoethanolamine (diacyl-PE) and mildly acidic amphiphiles, such as oleic acid or cholesteryl hemisuccinate, have been proposed among the first formulations, showing efficient delivery of diverse molecules to the cytoplasm [105]. In particular, 1,2-dioleoyl-PE (DOPE) forms a bilayer structure (Lα phase) at neutral pH, but when the pH is lowered under a certain threshold, a transition to the inverted hexagonal phase II (HII phase) occurs, causing membrane destabilization and cargo release [106]. However, the in vivo applications of these preparations is limited by their moderate stability and/or rapid removal by the mononuclear phagocyte system (MPS) after intravenous administration [107]. Therefore, to obtain clinically viable formulations, it is important to focus on serum-stability and in vivo half-life. Serum stability is most commonly obtained by incorporating into the bilayer structure a small amount of a PEGylated lipid, usually a *N*-(carbonyl-methoxypolyethylene-glycol-2000)-dyacil-PE (PEG2000-PE) or PEG5000-PE, depending on the length of the PEG moiety. (see Section 2). These stable formulations of liposomes can be endowed with a pH-induced uncovering and cargo release capability, covering them with pH-responsive polymers [108] whose protonation leads to destabilization of the bilayer lipid structure [109]. One of the first examples of this class of macromolecules are copolymers of *N*-isopropylacrylamide (NIPAM) and methacrylic acid (MAA) [110], in which the pH-sensitive moiety is the carboxylic group of MAA. Terminally alkylated NIPAM/MAA copolymer was used to cover PEGylated liposomes. The chosen PEGylated lipid, PEG5000-PE, efficiently increased the circulation time, did not impair pH sensitivity, and the properties were maintained after incubation in serum [109].

Ghanbarzadeh et al. [111] prepared pH-sensitive and plasma-stable liposomes using PEG-poly-(monomethylitaconate)-CholC6 (PEG-PMMI-CholC6) loaded with rapamycin. The deprotonated state of the carboxylic acid groups at neutral pH warrants the solubility of PEG-PMMI-CholC6 that precipitates at low pH because of the protonation of carboxylates, increasing the lipophilic character of the polymer. The consequent liposome surface destabilization leads to a rapid payload release. In vitro studies showed high stability at neutral pH with less than <10% of leakage in plasma after 3 h and efficient rapamycin delivery with more than 60% of cell inhibition upon lowering the pH to 6.5 in HT-29 cells.

**Figure 9:**
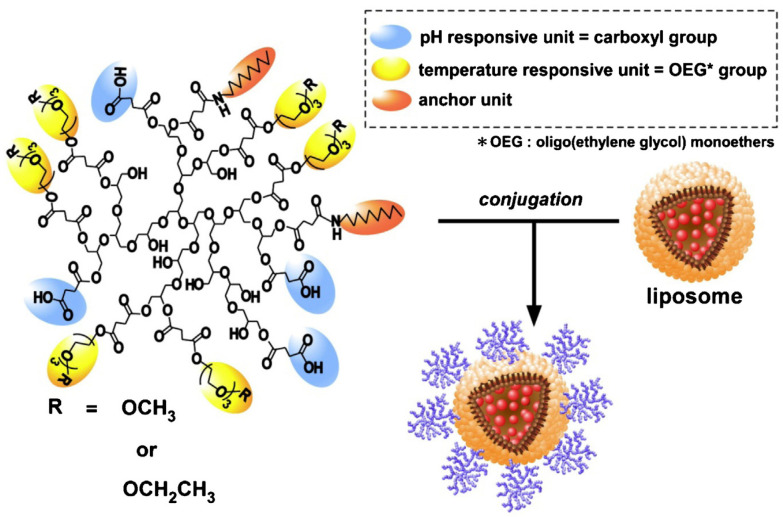
Design of temperature and pH dual-stimuli-responsive liposomes using hyperbranched poly(glycidol)s with temperature-sensitive OEG groups and pH-sensitive succinyl groups. Reproduced with permission from [112]. Copyright © 2014 Elsevier.

A dual-stimuli-sensitive biocompatible polymer was developed by Kono et al., based on hyperbranched poly(glycidol) (HPG) bearing temperature-sensitive oligo(ethylene glycol)s (OEGs) and pH-sensitive succinyl groups [112]. Liposomes of phosphatidylcholine covered with this polymer could be destabilized upon both a slight temperature increase (30–40 °C) and slight pH decrease (4.0–5.5) due to the protonation of carboxyl groups of the grafted polymer that changes its character from hydrophilic to hydrophobic (Figure 9). Liposome destabilization was also attributed to hydrogen bonds formation between the polymer carboxyl groups and the phospholipid head-groups. Simultaneous stimulation by low pH and high temperatures resulted in an enhanced cargo release than the temperature-induced one alone.

PSLs can also be prepared by the incorporation of protonable lipids within the liposomal membrane such as cholesteryl hemisuccinate (CHEMS) [113], which switches from negatively charged at neutral pH to neutral at acidic pH, thereby causing bilayer disruption. As already mentioned, PSLs are usually PEGylated. The PEGylated lipid can be inserted by mixing it in the lipid blend used for the liposome formation (pre-insertion method), resulting in PEG polymer protrusion from both sides of the bilayer. Alternatively, preformed liposomes can be incubated with different amounts of PEGylated lipid micellar solutions that insert in the bilayer (post-insertion method), leading to protrusion of the PEG polymer only on the exterior side of the liposome [114]. It is postulated that the viscosity of the inner lipid monolayer is reduced, leading to increased bilayer fluidity, facilitating the Lα-HII transition upon pH decrease. PSLs with post-inserted PEGylation show an enhanced pH-dependent release, suggesting that post-insertion PEGylation may offer advantages in terms of pharmacokinetics. Xu et al. proposed a polymeric derivative, poly(2-ethyl-2-oxazoline)-cholesterol hemisuccinate (PEtOz-CHEMS), to construct PSLs loaded with doxorubicin (DOX) [115], inserting the polymer using the post-insertion method [116]. These PEtOzylated liposomes (PEtOz-L) showed an acidic pH-induced size increase, a pH-dependent DOX release, and a better in vitro cellular uptake at pH 6.4 compared with conventional liposomes (CL), CHEMS-modified liposomes (CH-L), and PEGylated liposomes (PEG-L). It was demonstrated by confocal laser scanning microscopy images that PEtOz can help liposomes achieve the “endosomal escape” as the liposomes can fuse with the endosomal membrane thanks to the acidic conditions of endosome and release DOX into the cytoplasm. The in vitro cytotoxicity of PEtOz-DOX-L was found higher than those of CL-DOX, CH-DOX-L, and PEG-DOX-L under low pH conditions, confirming the pH-responsive PEtOz as a promising material for intracellular targeted delivery system.

The addition of cholesterol-terminated poly-(acrylic acid) (Chol-PAA) to liposome with the post-insertion method, followed by crosslinking with 2,20-(ethylenedioxy)-bis(ethylamine), forms a pH-sensitive polymer cage, inducing the release of two pharmacologically active types of cargo (namely As^III^ and Ni^II^) co-encapsulated in the liposomal core. The chemomechanical release property arises from the stimuli-responsive conformational change of the polymer cage, due to the protonation of the free acrylate groups at low pH, which in turn, perturbs the lipid membrane [117].

DPPC:cholesterol liposomes were grafted with a poly(isoprene-b-acrylic acid) diblock copolymer to produce PLS entrapping curcumin as a free drug and as a water-soluble inclusion complex with PEGylated tert-butylcalix [4] arene, which allows the drug to occupy both the phospholipid membranes and the aqueous core of liposomes [118]. In this case as well, cargo release was due to protonation-induced liposome destabilization. Bioassay data showed that PLS-curcumin was superior in terms of cytotoxic activity compared to both the free drug and curcumin embedded in non-pH-sensitive liposomes, efficiently inhibiting the viability and proliferation of resistant cell lines.

A slightly different approach was proposed by Huang et al. [119], in which the pH-induced membrane perturbation caused by the grafted polymer resulted in transmembrane channel formation, leading to the hydrophilic cargo release. The channel-forming polymer was poly(acrylic acid)-g-poly(monomethoxy ethylene glycol) (PAAc-g-mPEG), that spontaneously self assembles into liposomes when mixed with the cationic lipid, didodecyldimethylammonium bromide (DDAB), through cooperative electrostatic interactions. To prepare copolymer/DDAB complex assemblies, a solution of the copolymer at pH 8.9, where AAc units are completely dissociated, was added into a vial containing a dry DDAB film followed by overnight incubation under stirring. Upon decreasing the medium pH to 5.0, a particle swelling was observed, due to the reduced ionization of AAc residues, leading to a loss of both electrostatic interaction between DDAB and AAc moieties and hydrophobic association originally induced by electrostatic pairings. The vesicular membrane was more hydrated and permeable to water influx into the inner aqueous compartment, explaining the observed swelling. Nevertheless, the virtually unchanged diffraction peak at 2θ = 2.93° in the WXRD pattern with pH suggests that the ordered interpenetrated layer morphology is still retained, and the structural transition only occurs in localized regions. It is therefore postulated that the local unionized AAc residues found at pH 5.0 are re-orientated into the membrane, forming transmembrane channels.

Another example of PSL, this time triggered by a slightly alkaline pH, is described by Barea et al. [57,120]. The aim was to prepare drug-loaded liposomes for oral administration, targeted to the colon. Vesicle coating should hence resist the acidic pH of the gastrointestinal tract, preventing the ingress of bile salts, which would lead to premature drug release, and dissolve in the slightly basic pH of the colonic tract. To this aim, liposomes made of POPC/cholesterol 7:2 and containing the model drug 5-ASA were prepared by the thin film hydration method that produces multilamellar vesicles (MLV), subsequently resized by extrusion through membranes of decreasing pore size. The obtained nanocarrier was first coated with chitosan, able to reduce the intake of bile salts, and then entrapped in microspheres of the pH-responsive Eudragit S100, a methacrylic acid copolymer, using a double emulsion-solvent evaporation technique. In vitro drug release studies showed that drug release was prevented (>10%) within a simulated stomach and small intestine, while in the simulated large intestine, the Eudragit S100 coating was degraded, triggering the release (>85%) of the encapsulated drug. Subsequently, a green method for the Eudragit S100 liposome coating was proposed by De Leo et al. [20]. In detail, the model drug curcumin was encapsulated into small unilamellar vesicles (SUVs) of about 40 nm prepared by the micelle-to-vesicle transition method (MVT) starting from ethanol solutions. A coating with Eudragit S100 was obtained by dissolving the liposomes and the polymer in a slightly alkaline solution (phosphate 0.1 M pH 8.0) in which the polymer is soluble. This solution was quickly diluted to 1:10 in an acidic solution (acetic acid 0.25% *v/v* pH 3.5) that induces polymer precipitation around the liposomes, forming small clusters of micrometric dimensions. The Eudragit S100 covering could be easily dissolved at pH ≥ 7.0. Curcumin-loaded liposomes displayed the same antioxidant activity of free curcumin in ethanol, a negligible antioxidant activity when covered with the polymer, which reversed to the normal one after polymer removal. In a subsequent work [19], the uptake by Caco-2 cells of vesicles loaded with curcumin and coated with Eudragit S100 was assessed. At pH > 7.0, the Eudragit S-100 coating dissolves, releasing the nanometric liposomes and allowing them to enter Caco-2 cells. The curcumin released upon vesicles dissolution was then able to significantly decrease intracellular ROS levels induced by H_2_O_2_. In this way, the possibility of realizing gastroresistant liposome formulations was demonstrated for the delivery of antioxidant molecules to Caco-2 cells used as a model of intestinal epithelial cells.

**Figure 10:**
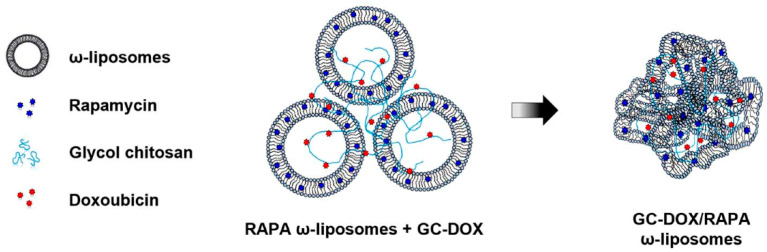
Schematic illustration of liposome containing doxorubicin and rapamycin and complexed with glycol chitosan. The nanovectors are stable at pH 7.4 and aggregate when the pH is lowered to 5.0. Reproduced under Creative Common license from ref [121].

PSL embedding both the hydrophobic rapamycin (RAPA) and the hydrophilic doxorubicin (DOX) were also prepared using glycol chitosan (GC) to achieve the pH-triggered drug release profile [121] (Figure 10). To this aim, ω-liposomes made of docosahexaenoic acid (DHA) and loaded with RAPA were first prepared by the thin-lipid-film hydration method. GC and DOX where covalently bound together, forming an amide bond after activation with EDC (1-ethyl-3-(3-dimethyl aminopropyl) carbodiimide) and NHS (*N*-hydroxy-succinimide). Finally, GC-DOX/RAPA ω-liposomes were prepared by dissolving GC-DOX in deionized water (pH 6.5) and adding it dropwise to a RAPA ω-liposome solution under stirring, forming a precipitate that was collected by centrifugation. The complexation is driven by the electrostatic interaction between the liposomes, which have a strong negative surface charge, and glycol chitosan, which has oppositely charged surface amine groups. RAPA ω-liposomes were 90 nm in diameter with a −30 mV surface charge, increasing to 130 nm upon complexation with GC-DOX at a ratio of 20:1, while the surface charge was partially neutralized to −15 mV. To evaluate their pH stability, GC-DOX/RAPA ω-liposomes were incubated at various pH values (pH 4.0, 5.0, 6.5, or 7.4). The particle size was stable near pH 7.4 and 6.5, while aggregation of the particles started from pH 5.0 due to the decrease in surface charge because the amine group of GC is protonated by hydrogen ions under acidic conditions. The neutralization of the surface charge forces particles to interact with each other, disrupting the structure of the particles and leading to cargo release.

pH-Temperature dual-sensitive liposomes (CPTLPs) were obtained as an efficient drug delivery system [122] exploiting the temperature sensitivity of 1,2-dipalmitoyl-sn-glycero-3-phosphocholine (DPPC) and the pH sensitivity of polyaspartic acid (PASP) grafted with octylamine (PASP-g-C8). The resulting nanovector showed improved targeting and availability of liposomes to cancer cells. Liposomes made of cholesterol and cationic temperature-sensitive lipids, loaded with the model drug Cytarabine (CYT), were first prepared and covered with pH-sensitive PASP-g-C8 using octylamine for anchoring. The zeta potential of CPTSLs was −42 mV, suggesting a stable colloidal system. CPTLPs remained active in both normal tissues (with pH 7.4 and 37 °C) and tumor tissues (with pH 5.0 and 42 °C) and showed significant pH-temperature sensitivity and a more prolonged release than control groups. MTT tests indicated that the cell apoptotic effects induced by liposomal CTY compared with free CTY were nearly 30% higher in HepG2 cancer cells, and 20% lower in healthy cells.

## 6. Polymer/Liposome Assembly to Provide Targeting Platform

Targeting ligands for the functionalization of the vesicles can be implemented in the formulation of liposomes in order to increase their application potential in the field of drug delivery, from cancer therapy to bacterial biofilm and fungal infection treatment, and from gene delivery to applications for vaccines [36,123,124]. An appropriate polymeric functionalization or a polymeric coverage can be used to obtain liposomes capable of reaching their target through a passive or active strategy. In Section 2, we illustrated how a PEG coating can provide stabilization of vesicles in biological fluids and, in particular, increase their residence time in the bloodstream. This is achieved by avoiding interaction with serum proteins and avoiding the recognition and elimination mechanisms that constitute the body’s first line of defense. As cancerous tissues have enhanced permeability and limited lymphatic drainage, long circulating liposomes can accumulate there similarly to other macromolecules. This effect is called the enhanced permeability and retention (EPR) effect [125] and, therefore, the PEGylation of liposomes can be considered a passive targeting strategy. It has been observed that the improved solubility together with the decreased liposome aggregation induced by PEG leads to a 10 times longer circulation time and increases liposome accumulation into the target tissues [126,127,128]. The dimensions of the liposomal carrier proved to be crucial in determining the extent of this effect, which seems to be maximized for diameter values less than 200 nm [129].

Alternatively, it is possible to adopt an active targeting strategy, which allows the binding of the lipid carrier in a selective way on its target, releasing its payload in the tumor (or pathological) microenvironment and therefore increasing the effectiveness of the therapeutic treatment and, at the same time, lowering drug doses, reducing side effects. The active targeting of liposomes involves the grafting of a targeting ligand to the surface of vesicles, capable of recognizing a specific receptor expressed by the cellular target. Such a targeting ligand can be noncovalently incorporated in the bilayer or most commonly covalently linked to lipids or to the distal end of polymers anchored to the bilayer, commonly PEG chains of PEG modified lipids. These lipid building blocks, functionalized with the desired ligand, can be added to the lipid formulation in the first step of liposome preparation, or they can be grafted via (mixed) micelles to the preformed liposomes [130,131].

**Figure 11:**
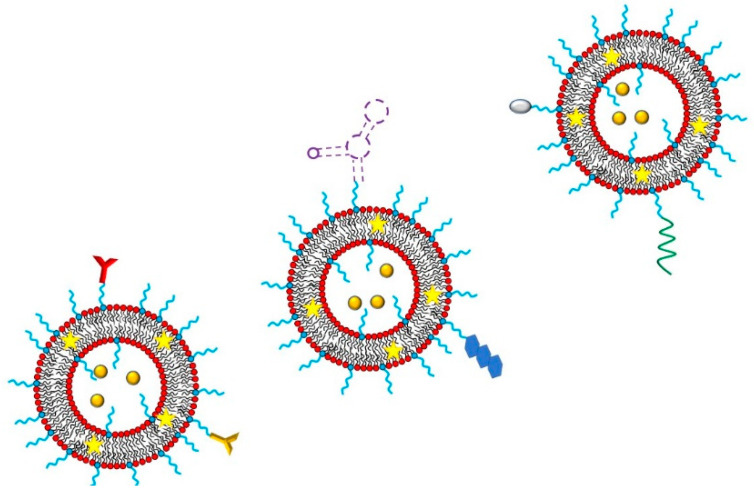
Schematic representation of targeted liposomes, with targeting ligands covalently linked to the distal end of PEG-lipids. For illustrative purposes, antibodies are shown on the left, aptamer and sugar in the middle, and a generic small molecule and peptide are sketched on the right.

The commonly used targeting ligands are antibodies, proteins, peptides, carbohydrates, aptamers, and small molecules capable of being recognized by specific cell surface proteins or receptors on cancer cells [132,133] (Figure 11).

The PEGylated lipids used in the formulation of the targeted liposomes have the PEG chain that ends with a suitable functionality, capable of forming a covalent bond with a functionality present on the ligand. PEGylated lipids with these characteristics can be synthesized ad hoc but are now widely commercially available. For example, PEGylated lipids are available, which terminate with an amino group or a carboxylic group, useful for the formation of an amide bond with the ligand; or ending with a maleimide group useful for the formation of thioether bonds by reaction with a thiol group, or groups suitable for “click chemistry” reactions.

At the same time, the polymers used for other specific functions can perform targeting functions, confirming the great versatility of liposomal systems. This is the case for hydrophilic polysaccharide HA, used as a backbone of the multifunctional pH-responsive polymers. Recently, Miyazaki et al. designed HA-based pH-sensitive polymer-modified liposomes having not only pH-sensitivity but also targeting properties to cells expressing CD44, which is known as a cancer cell surface marker [120].

## 7. Conclusions and Perspectives

Liposomes are very versatile carriers capable of satisfying many of the experimental needs that arise in the field of drug delivery. The properties of first-generation phospholipid vesicles can be expanded thanks to the possibility of grafting both natural and synthetic polymeric molecules into the bilayer, to obtain second- and third-generation vesicles with increased stability, load capacity, and ability to respond to external stimuli or to actively recognize a specific target. Alternatively, the liposomes can be incorporated in a suitable polymeric matrix capable of responding to stimuli or acting as a deposit of drugs and bioactive molecules.

In this review, a representative overview of the various manufacturing and application possibilities in the field of polymer/liposome assembly was presented, with a look at the most recent literature, without however omitting the fundamental concepts.

Research in this sector is active both in the synthesis of new polymeric materials, as well as in the creation of new architectures and in the supramolecular organization of the various building blocks, as evidenced by the vast literature available in the various databases.

The challenge in the future will be to expand the basket of commercially available materials (amphiphilic polymers, modified lipids, responsive molecules, etc.), while at the same time, containing the costs that today constitute the bottleneck for the scaling-up of these innovative materials and their real clinical applications.
